# Microscopic Image of the Protoscolex of *Echinococcus granulosus* on the “Hydatid Sand”

**DOI:** 10.4269/ajtmh.2010.09-0743

**Published:** 2010-06

**Authors:** Antonio Soriano Arandes, Frederic Gómez Bertomeu, Joaquin Maldonado Artero

**Affiliations:** Department of Pediatrics, University Hospital Joan XXIII, Tarragona, Spain; Department of Microbiology, University Hospital Joan XXIII, Tarragona, Spain; Department of Pediatric Surgery, University Hospital Joan XXIII, Tarragona, Spain

**Figure 1.**
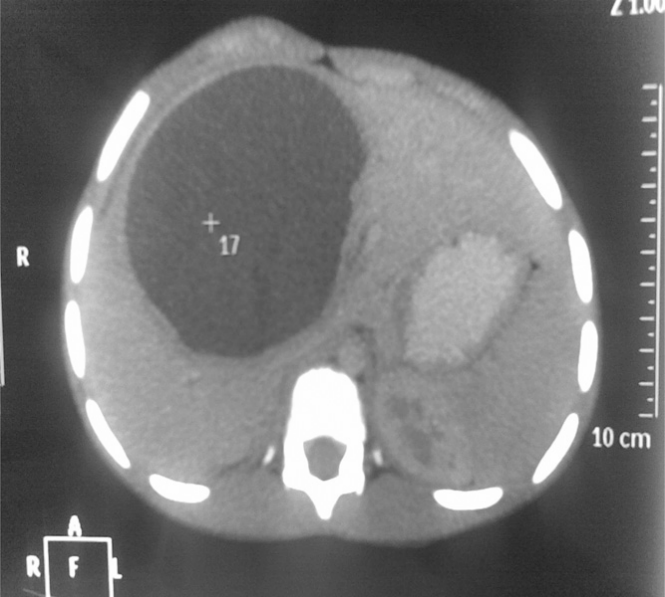
Image from a computed tomography study of the abdomen of the patient.

**Figure 2.**
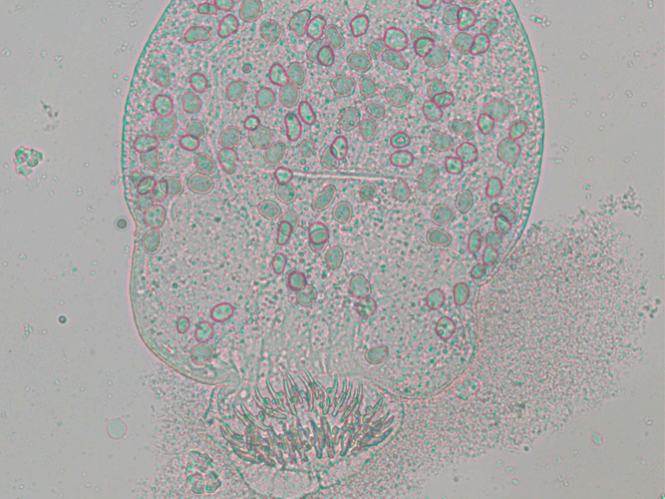
Hydatid sand containing a protoscolex of *Echinococcus granulosus* was seen by light microcopy. This figure appears in color at www.ajtmh.org.

An 8-year-old child originally from Morocco spent 1 month in Spain before complaining of abdominal pain for the previous 24 hours. There was no fever, vomiting, or diarrhea. He had lived in a rural area with dogs and sheep. Physical examination detected a mass in upper-right abdominal area. Chest X-ray showed right hemidiaphragmatic elevation. Ultrasonography showed a hypoechoic cyst mass of 12.5 × 10.5 × 8.5 cm without septum or calcifications that was a type CE1 according to the World Health Organization standardized classification.^1^,^2^ Computed tomography (Figure 1) and magnetic resonance imaging confirmed the diagnosis of hepatic hydatid cyst. Invasion into the biliary tree bile ducts, portal vein, or hepatic vein was not seen. IgE-specific serology for *Echinococcus granulosus* was positive (18.3 kUI/L). Serology for *Entamoeba histolytica* was negative. Aspartate and alanine amino transferase levels were increased (145 U/L and 151 U/L, respectively). Treatment with albendazole was initiated, and cystectomy was performed on the 18th day after admission. Gross examination showed hydatid sand; the protoscolex of *E. granulosus* was seen by light microscopy (Figure 2). Albendazole was maintained for 3 months and discontinued when ultrasound and serology confirmed resolution. The patient remained asymptomatic on follow-up visits.
